# The BJGP Open Top 10 Most Read Research Articles of 2023: an editorial

**DOI:** 10.3399/BJGPO.2024.0042

**Published:** 2024-02-29

**Authors:** Alexander Burrell, Hajira Dambha-Miller

**Affiliations:** 1 BJGP Open, Royal College of General Practitioners, London, UK

**Keywords:** editorial, primary healthcare, general practitioners

At BJGP Open, we are proud to be a journal for international primary care, early career researchers, and front-line clinicians. In 2023, we accepted more articles than ever before and exceeded our previous monthly submission numbers towards the end of the year. We published works from 17 different countries (based on lead authors), and their submissions represented a diverse range of clinically and socially relevant primary care topics. This included manuscripts examining the driving forces behind GP migration in Europe; the impact of GP training opportunities on health inequities; and the challenges faced by international medical graduates in primary care. In this editorial, we explore those works which have made it onto our list of *Top 10 Most Read Research Articles of 2023*.

Remote consulting and telehealth remain high on the research agenda. The COVID years ushered in a new era of remote consultations and, while we continue to see submissions on this topic, authors are now looking to the future, examining the sustainability and impact of this approach. Ashley *et al* ^1^ thoroughly explored this in Australia through their qualitative study interviewing a range of clinicians, including GPs, nurses, nurse practitioners, and allied health professionals. The authors report that remote consultations are sustainable going forwards, but standards, training, and hybrid models need development. Concerns around the safety and effectiveness of remote consulting were explored by Lane *et al* ^2^ in their analysis of 281 in-person consultations on diabetes and cardiovascular disease: 60% of clinical tasks were ‘easily or relatively easily’ translatable to telehealth, with a further 26% ‘moderately translatable’ if patients acquire their own equipment, such as blood pressure machines. Can we expect all patients to accept this, and what happens to the most vulnerable? Verity and Tzortziou Brown’s^3^ article addresses this question by exploring the perspective of individuals from inclusion health groups on remote consulting. Potential barriers highlighted include issues around digital exclusion, availability of language translation services, and challenges navigating an already complex healthcare system. The economic impact of remote consulting also received consideration through Anthony *et al*’s^4^ feasibility economic analysis of ‘ThinkCancer!’, a complex behaviour change intervention to improve the timely diagnosis of cancer. The study reports successful remote delivery of the intervention and follow-up at an average cost of £1317 per practice. Sufficient data were collected to inform future definitive economic evaluation, reminding us how both clinical and academic communities were able to respond in challenging times.

For research to be translated into GP practices, understanding clinicians’ experiences is key. Mizumoto *et al* ^5^ explored GP perceptions of being asked to address social determinants of health in clinical settings: while participants were aware that addressing these issues would enhance their practice, being mandated to ask set questions was not useful in a busy and often isolated primary care setting. Guidelines are just that, and are used or adapted by clinicians based on their real-world experience. Jones *et al*’s^6^ systematic review of challenges faced by GPs when providing palliative care in the UK found a number of barriers: a fragmented multidisciplinary team approach, challenging communication with patients and carers, and inadequate training to address the complexities of palliative care. Through understanding the reality of delivering clinical care, we can identify areas to focus our academic and quality improvement efforts. Donaghy *et al* ^7^ evaluated the ‘Living Well Assessment’ quality improvement project, a GP-led comprehensive geriatric assessment in primary care conducted in ten practices during the pandemic. This was found to be feasible and highly valued by both frail patients and GPs, though the authors note further investigation is required regarding efficient use of GPs' time, and clinical and cost effectiveness: we look forward to reading these studies.

Once clinical practice is established, it is important to remain reflexive and to examine why we do what we do. Borek *et al* ^8^ qualitatively explored clinician and patient views on hypothetical advice to stop antibiotics when feeling better rather than completing a pre-set course for urinary tract infections. Clinicians were more open to this than patients, but both groups felt good evidence and guidelines were needed and these decisions should be shared. Jones *et al* ^9^ investigated variation in laboratory testing for patients with hypertension, type two diabetes, and chronic kidney disease. They found considerable variation by practice, indicating uncertainty over most appropriate testing frequencies. Time is a valuable commodity in primary care, and both over- and under-testing can affect how much of it we have. Dahle *et al* ^10^ may have found a way to save a minute or two: the single question Emoqol-100 seems to have high validity in diagnosing depression when compared to longer multi-item tools.

In our 2022 editorial,^11^ we were looking forward to submissions building the evidence base on remote consulting: our top articles from 2023 have started to do this and have also provided valuable insights into the realities of clinical care from a range of perspectives and environments globally. Evaluating practice that has been doctrine, such as antibiotic course length or chronic disease monitoring frequency, can highlight areas for improvement. As a journal, we are looking forward to receiving submissions on these and other important topics as we continue to work with our authors to improve and develop the international primary care evidence base.
